# Simultaneous versus Sequential Cessation of Tobacco and Cannabis: Preferences of Young Adults Who Co‐Use in France

**DOI:** 10.1111/dar.70221

**Published:** 2026-08-09

**Authors:** Tangui Barré, Géraldine Cazorla, Vincent Leroy, Gwenaëlle Maradan, Clotilde Couderc, Julia de Ternay, Carlotta Magnani, Patrizia Carrieri

**Affiliations:** ^1^ Aix Marseille Univ Inserm, IRD, SESSTIM, Sciences Economiques & Sociales de la Santé & Traitement de L'information Médicale, ISSPAM Marseille France; ^2^ ORS PACA Southeastern Health Regional Observatory Marseille France; ^3^ Association Addictions France Paris France; ^4^ Service Universitaire d'Addictologie de Lyon, Hôpital Édouard Herriot, Hospices Civils de Lyon Lyon France; ^5^ Research on Healthcare Performance RESHAPE, INSERM U1290 Université Claude Bernard Lyon 1 Villeurbanne France

**Keywords:** cannabis, co‐use, sequential cessation, simultaneous cessation, tobacco

## Abstract

**Introduction:**

Co‐use of tobacco and cannabis is common among young adults and is associated with higher dependence and quitting difficulties. Little is known about the preference for simultaneous or sequential dual cessation in this population. We aimed to explore preferences for the timing of dual cessation and the methods most likely to be used by young adults who co‐use.

**Methods:**

Data came from an online nationwide cross‐sectional survey of people co‐using tobacco and cannabis in France. Preferences for the timing of dual cessation were modelled using binary logistic and multinomial regressions among participants aged 18–30 years. We also described cessation methods (for tobacco and cannabis separately) previously tried and those considered likely to be used in future quit attempts.

**Results:**

Among 357 young adults (54.9% men; median age 24), those using tobacco daily and cannabis ≥ 20 days/month had a 56% lower likelihood of preferring simultaneous over sequential cessation (adjusted odds ratio 0.44 [0.21–0.90], *p* = 0.024) compared with participants with the least frequent co‐use. Participants who perceived their tobacco use as more harmful than their cannabis use were more likely to prefer quitting tobacco first. Across past and future attempts, the most cited methods for both substances were sport, cannabidiol, electronic cigarettes and nicotine replacement therapy.

**Discussion and Conclusions:**

Simultaneous cessation appeared more acceptable to young adults with lower‐risk co‐use patterns. Overall, the likelihood of using recommended cessation methods was low. Further research is needed to develop effective and acceptable sequential cessation strategies for young adults who co‐use these substances.

## 1 Introduction

In 2019, tobacco smoking represented the primary global risk factor for premature death among men [1]. Although its prevalence has declined since 1990 [2], smoking still accounted for 10%–20% of disability‐adjusted life years in most European countries in 2019 [1], with France showing a particularly high smoking burden [3]. In France in 2024, 17.4% of adults aged 18–79 smoked tobacco daily, a proportion similar to that observed among those aged 18–29 (18.4%) [4].

Young adulthood is a critical period for establishing and reinforcing tobacco smoking as a stable, long‐term habit [5, 6, 7]. During this stage, cannabis co‐use has been identified as a major risk factor for progression to sustained and frequent tobacco smoking [6, 8]. Among adolescents and young adults, cannabis use has also been associated with poorer academic achievement and increased mental distress [9, 10].

According to the most recent surveys, 15.0% of EU residents aged 15–34 reported cannabis use in the past year [11]. In contrast to tobacco, the prevalence of cannabis use in Europe increased between 2010 and 2019 [12]. However, the burden of cannabis use disorder (CUD) has not shown a parallel rise, with age‐standardised disability‐adjusted life years rates remaining high but relatively stable in Western Europe, particularly among young adults [13, 14]. In 2023 in France, where cannabis possession remains illegal, past‐year prevalence exceeded 10% among adults aged 18–64, and 6.6% of those aged 18–24 reported regular use (defined as 10 or more uses in the past month) [15]. In 2021 in France, 80% of adults who used cannabis in the last month also smoked tobacco cigarettes daily. The prevalence of tobacco and cannabis co‐use (defined as daily cigarette smoking and at least monthly cannabis use) was 8.3% among adults aged 18–30, and adults aged 18–30 represented 46.3% of adults who co‐used [16].

People who co‐use tobacco and cannabis—defined as using both substances either separately or simultaneously within a recent period—represent a key target for reducing substance‐related harms [17], as the two substances are closely interconnected in multiple ways [18]. For instance, cannabis is commonly mixed with tobacco in joints [19, 20, 21]. People who co‐use face heightened tobacco‐related risks, as they tend to smoke more frequently, exhibit greater nicotine dependence [22, 23, 24, 25], and achieve poorer tobacco cessation outcomes [26, 27, 28, 29, 30]. In addition, tobacco co‐use may increase the likelihood of developing CUD [31], and the combined use of both substances leads to greater toxicant exposure [32, 33]. Dual cessation among people who co‐use, especially young adults, is therefore highly recommended but also very challenging [34], and young adults who co‐use may have limited recourse to formal care and to evidence‐based, recommended interventions [35].

Yet, little is known about the most efficient approach to dual cessation. On one hand, simultaneous cessation (i.e., quitting tobacco and cannabis at the same time) may lead to heightened, albeit transient, withdrawal symptoms [36], thereby impeding successful cessation [37]. Deciding to attempt simultaneous cessation may also require greater willpower, as quitting two substances at once may appear more difficult than taking a step‐by‐step approach. On the other hand, sequential cessation (i.e., quitting one substance and then the other) may maintain exposure to smoke‐related cues, thereby precipitating relapse for the substance quit first.

Most studies on tobacco‐cannabis dual cessation have implemented simultaneous treatment approaches and found them to be acceptable [34]. In their controlled proof of concept, Lee et al. compared a simultaneous versus sequential tobacco intervention among adults (mean age of 34) seeking treatment for CUD [38]. They reported poor tobacco cessation outcomes that did not differ between arms.

Sharing decision‐making regarding substance use disorder treatment is important, and embracing patients' preferences may help reduce substance use. In a qualitative study among young adults who use both electronic cigarettes and cigarettes, a heterogeneity was observed in preferences for the timing and order of products to quit. A similar heterogeneity may be expected for tobacco and cannabis co‐use.

This question of preferences also extends to methods for quitting and needs to be studied in this specific group of young adults. For instance, regarding tobacco cessation, it has been reported that, compared to older adults, young adults are less likely to make assisted quit attempts or to use pharmacotherapy [39, 40, 41, 42]. This observation suggests that the range of available cessation methods does not align with the specific preferences of this group, despite its high need for support. Moreover, preferences for timing and methods may influence each other, as some methods may facilitate quitting both substances by targeting shared triggers of use (e.g., stress‐relief‐based methods), while others may be substance‐specific (e.g., nicotine replacement therapy). To develop interventions that are more likely to achieve high adherence, it is therefore necessary to understand which types of cessation approaches young adults who co‐use tobacco and cannabis would prefer, depending on their co‐use patterns.

The present study aimed at identifying patterns of tobacco and cannabis co‐use associated with preferences for simultaneous or sequential cessation among young adults, and to examine their preferred types of methods for quitting both substances.

## 2 Methods

### 2.1 Design

The TOBASCO study (TOBAcco‐cannabiS CO‐use in young adults) was based on an online cross‐sectional survey conducted throughout France between 1 July 2024 and 1 July 2025, with no compensation provided to participants [43]. Recruitment relied on a variety of national dissemination strategies, including: (i) social media groups and pages dedicated to addictive behaviours; (ii) online platforms linked to cannabis‐related publications; (iii) mailing lists and social media accounts of student associations; (iv) local youth support services (*missions locales*) through online posts, posters and leaflets; (v) university health services via targeted poster and flyer distribution; and (vi) the professional networks of the research team. Additionally, posters and flyers containing a QR code giving direct access to the survey were displayed in 16 addiction prevention and treatment facilities. To further strengthen participation, a professional survey company also organised a one‐day face‐to‐face recruitment campaign in a public space in Paris in April 2025.

The study protocol complied with the ethical standards of the Declaration of Helsinki and was approved by the ethics committee of Aix‐Marseille University (IRB00014113, 2023‐12‐14‐01; 14 December 2023). Informed consent and authorisation for personal data processing were obtained from all participants prior to survey completion. For data privacy reasons, IP addresses were not collected. Consequently, no check for duplicate responses was performed.

### 2.2 Participants

The TOBASCO study focused on participants aged 18 to 30 who had consumed both tobacco and cannabis within the previous 7 days. Co‐use eligibility was established at the beginning of the survey through two separate screening questions, one for each substance: ‘Have you smoked [tobacco/cannabis] in the past 7 days (either alone or mixed)?’ (Yes/No). In this study, co‐use was thus defined as the combustion‐based use of both tobacco and cannabis in the past week, whether used simultaneously or separately. In addition to combustion‐based use, some participants may have also employed smoke‐free methods to administer these substances or related products. Only individuals who completed the entire questionnaire were included in the analytical sample.

### 2.3 Data Collection

The average duration to complete the questionnaire was 16 min. Socioeconomic and demographic data collected included gender, age, region of residence, educational level and self‐perceived economic situation. The latter was assessed using the question: ‘Presently, would you say that, financially speaking …’ with six response options ranging from ‘You are very comfortable’ to ‘You cannot manage without going into debt’.

The questionnaire collected the number of days of use per month (tobacco and cannabis separately); nicotine dependence using the Fagerström Test for Nicotine Dependence [44] (FTND, administered to participants reporting ≥ 25 days of tobacco smoking per month; otherwise, the absence of dependence was assumed), CUD using the 3‐item CUDIT‐Short Form (CUDIT‐SF, administered to all participants) [45] (hereafter referred to as ‘dependence’ for consistency); and electronic cigarette use (Yes, but not daily/Yes, daily/No). The decision to administer the FTND questionnaire only to participants smoking at least 25 days per month was made to minimise questionnaire completion time. This choice is justified by the fact that intermittent smokers have a low likelihood of nicotine dependence [46] and typically achieve low FTND scores [47]. Among light smokers, the FTND provides little additional information beyond the number of cigarettes smoked per day [48]. Indeed, the FTND was originally developed using a sample of individuals smoking an average of one pack per day [44], and its questions are therefore tailored to daily smokers. Participants were also asked whether they mixed cannabis with tobacco when smoking it (Always/Sometime/Never), whether, during a single occasion, they ever used cannabis without tobacco or tobacco without cannabis (Never or almost never/Sometimes/Always or almost always), and whether they knew about vaporizers (No/Yes, but no past‐month use/Yes, past‐month use). Participants who used electronic cigarette were asked whether their current use was aimed at reducing or quitting [tobacco/cannabis] (Yes/No).

History of tobacco and cannabis cessation attempts was collected through three questions: “In your lifetime, how many attempts have you made to quit for at least 48 h […]?” regarding (i) tobacco (while continuing to use cannabis); (ii) cannabis (while continuing to use tobacco); (iii) both tobacco and cannabis simultaneously. Response options were: Zero/One or two attempts/Three to five attempts/6 to 10 attempts/More than 10 attempts. Participants were also asked whether they currently wished to reduce their tobacco or cannabis use (Yes/No).

Participants were asked whether they were currently receiving care for an addictive behaviour (Yes/No) and, if so, for which substance or behaviour (five non‐exclusive options).

Two questions assessed participants' relative perceptions regarding tobacco and cannabis use: (i) ‘Which substance do you perceive yourself as being most dependent on?’ (Tobacco/Cannabis/Both equally/I am not dependent on either substance); and (ii) ‘According to you, based on your current patterns of use, which substance do you think poses the greatest risks to your health (physical or mental)?’ (Tobacco/Cannabis/Both equally/I do not know).

Regarding preferred types of methods, two types of questions were asked for both substances. First, participants who reported at least one quit attempt for a given substance were asked: ‘For your [tobacco/cannabis] quit attempts (past or ongoing), what have you tried?’. They then indicated which strategies they have tried (Yes/No) from a predefined list of 13 non‐exclusive options (11 for cannabis, as nicotine replacement therapies and the vaporizer were only proposed for tobacco). Second, all participants were asked: ‘If you were to stop using [tobacco/cannabis] in the coming month, would you be likely to turn to …’. The same predefined lists were provided, with three possible answers (Not at all/Somewhat/Very).

The predefined strategies were: nicotine replacement therapy; prescription medications; dietary supplements, over‐the‐counter medications and related products; electronic cigarette; cannabidiol; consulting a healthcare professional for follow‐up; consulting another professional for follow‐up; group and/or remote support; relaxation/meditation; hypnosis; sports; vaporizer (to decouple cannabis use from tobacco use); and family therapy.

This list included methods recommended by the French National Authority for Health for tobacco cessation (nicotine replacement therapy; prescription medications; consulting a healthcare professional for follow‐up, and, to some extent for specific populations, electronic cigarette) [49]. It was supplemented by methods not recommended by this institution considered low‐risk and for which there is no reason to discourage patients from using them (physical activity—renamed as “sports” to sound more familiar to young adults; and hypnosis) [49]. “Group and/or remote support” was included, as it is commonly featured in public health programmes such as “Stoptober” (adapted as “Mois sans tabac” in France) [50]. Candidate interventions currently under study, which have not yet demonstrated sufficient evidence of efficacy for tobacco or cannabis cessation, were also included (relaxation/meditation [51, 52]; cannabidiol [53, 54]; dietary supplements [55]; vaporizer [56]). Given that young adults may be less likely to receive advice from healthcare professionals for quitting [57], we assessed whether they would be likely to turn to other professionals. Finally, based on services available in clinical practice and the authors' experience, family therapy was also included.

### 2.4 Outcomes

The primary outcome was the preference for one type of cessation, assessed through the following question: “If you had to quit both tobacco and cannabis in the coming month, would you prefer to quit:” (Simultaneous cessation/Tobacco cessation then cannabis cessation/Cannabis cessation then tobacco cessation).

Secondary outcomes were the likelihood of using each type of method for future cessation attempts (Somewhat or very likely vs. Not at all).

### 2.5 Explanatory Variables

The following variables were tested as main explanatory variables of preference for simultaneous or sequential cessation: (i) tobacco and cannabis use frequency were dichotomised (daily vs. non‐daily for tobacco; daily or near‐daily, i.e., ≥ 20 days per month [58] for cannabis), and a four‐modality variable combining both was created; (ii) self‐assessed relative dependence and self‐assessed relative risk perception (four‐modality variables); and (iii) mixing cannabis with tobacco (Always vs. Not always). Lastly, tobacco and cannabis dependence binary variables (i.e., FTND ≥ 4 and CUDIT‐SF ≥ 2 [44, 45]) were combined into a four‐modality dependence variable.

Tobacco use frequency was dichotomised using a 30‐day‐per‐month threshold, as daily tobacco use is a well‐established marker for tobacco‐related behaviours [59, 60, 61]. Moreover, people who smoke tobacco on a non‐daily basis are more likely to deny being smokers [62, 63, 64, 65], exhibit distinct perceptions of addiction and quit attempts compared to daily smokers [66]. Daily smoking thus represents a robust, easily assessable indicator that facilitates cross‐study comparisons and holds clinical relevance.

For cannabis use, no consensus exists to define intensive patterns. We therefore adopted the EMCDDA's international monitoring recommendation (≥ 20 days of use per month) [67], also endorsed by the International Cannabis Toolkit [58]. This “near‐daily” threshold—beyond ensuring harmonisation—effectively captures frequent use [68, 69], while avoiding the overly restrictive daily‐use criterion, which risks being met by too few individuals.

### 2.6 Statistical Analyses

Study sample characteristics were compared according to participants' preference for simultaneous or sequential cessation (simultaneous/tobacco first/cannabis first) using chi‐square test for categorical variables and Kruskal‐Wallis test for numerical variables.

A binary logistic regression model was performed with “preferring simultaneous cessation” as outcome, and gender, age, tobacco and cannabis use frequency, relative dependence, relative risk perception, and mixing practices as explanatory variables. An alternative binary model was also tested, with the four‐modality dependence variable instead of the frequency variable.

A multinomial regression analysis was subsequently performed with preference for simultaneous or sequential cessation as outcome (simultaneous/tobacco first/cannabis first), using the same adjustment variables as previously. An alternative multinomial model was also tested, with the four‐modality dependence variable instead of the frequency variable. Marginal means were estimated for the explanatory variables, and predicted probabilities of the three possible preferences were plotted against each modality of the explanatory variables. Predicted probabilities were compared pairwise across outcome categories within each level of the explanatory variable, and across levels of the explanatory variable within each outcome category (Wald test), with Bonferroni correction applied to adjust for multiple testing.

In a sensitivity analysis, we ran the same logistic and multinomial regression models after excluding participants reporting being in treatment for their tobacco or cannabis use.

The proportion of participants who have tried the predefined types of methods for quitting each substance was described among those who had ever made any attempt. We tested whether participants who used electronic cigarettes (daily or not) were more likely to have used them for a prior quit attempt using a chi‐square test. For each of the 11 methods available for both tobacco and cannabis, polychoric correlations were computed, and ρ and Bonferroni‐adjusted *p*‐values were reported.

Participant's putative future preferred types of methods for quitting each substance (“slightly” or “very” likely vs. “not at all”) were compared according to their preference for simultaneous or sequential cessation (three‐modality variable) using chi‐square tests. Paired comparison (simultaneous vs. tobacco first; simultaneous vs. cannabis first; tobacco first vs. cannabis first) were also performed using chi‐square tests with Bonferroni correction. We tested whether participants who used electronic cigarette (daily or not) were more likely (“slightly” or “very”) to use them for a future quit attempt using a chi‐square test. We also summed the number of types of methods declared as likely or very likely, and compared those numbers according to cessation preference (Kruskal‐Wallis test). The percentage of participants reporting none (i.e., ticking “not at all” for all methods) was compared for tobacco and cannabis cessation (McNemar test), as well as according to participants' cessation preference (chi‐square test).

All analyses were performed using Stata software version 17.0 for Windows (StataCorp LP, College Station, TX, USA).

## 3 Results

### 3.1 Study Sample Characteristics

Among the 357 participants, 54.9% were men. The median age was 24 years [interquartile range 21; 27]. Nearly half (47.6%) of the participants were recruited via social media, 8.7% through personal invitation, 7.6% through healthcare providers and 36.1% through other means. Simultaneous cessation was preferred by 29.7% of the sample, tobacco cessation first by 38.4% and cannabis cessation first by 31.9% of the sample. Compared with men, women were more likely to prefer quitting cannabis first rather than tobacco first (*p* = 0.007). The men/women difference for choosing cannabis first vs. simultaneous was also close to significance (*p* = 0.061). Additional participants' characteristics are provided in Table 1. Among participants who used electronic cigarettes, 61.0% reported using them to reduce or quit tobacco use (vs. 20.3% for cannabis).

**TABLE 1:** Study sample's characteristics according to their preference for simultaneous or sequential cessation (*n* = 357).

	Whole study population	Simultaneous	Tobacco first	Cannabis first	
	*N* (%)	*N* (%)	*N* (%)	*N* (%)	*p*‐value^a^
Age, in years (median [IQR])	24 [21;27]	24 [22;26]	24 [21;27]	24 [22;26]	0.906
Gender					0.019
Men	196 (54.9)	62 (58.5)	82 (59.9)	52 (45.6)	
Women	147 (41.2)	42 (39.6)	46 (33.6)	59 (51.8)	
Other/Do not want to answer	14 (3.9)	2 (1.9)	9 (6.6)	3 (2.6)	
≥ upper secondary school certificate	219 (61.3)	71 (67.0)	84 (61.3)	64 (56.1)	0.256
Being a student	112 (31.4)	36 (34.0)	44 (32.1)	32 (28.1)	0.624
Self‐perceived financial difficulties					0.854
It's difficult to make ends meet/You can't manage without going into debt	103 (28.9)	30 (28.3)	37 (27.0)	36 (31.6)	
You just get by	104 (29.1)	29 (27.4)	44 (32.1)	31 (27.2)	
You are very comfortable/You are comfortable	150 (42.0)	47 (44.3)	56 (40.9)	47 (41.2)	
Frequency of use^b^					0.021
None	58 (16.2)	25 (23.6)	21 (15.3)	12 (10.5)	
Daily tobacco use	63 (17.6)	21 (19.8)	20 (14.6)	22 (19.3)	
DND cannabis use	43 (12.0)	13 (12.3)	22 (16.1)	8 (7.0)	
Daily tobacco and DND cannabis use	193 (54.1)	47 (44.3)	74 (54.0)	72 (63.2)	
Dependence^c^					0.013
None	60 (16.8)	28 (26.4)	22 (16.1)	10 (8.8)	
Tobacco dependence	25 (7.0)	3 (2.8)	10 (7.3)	12 (10.5)	
Cannabis dependence	184 (51.5)	51 (48.1)	73 (53.3)	60 (52.6)	
Tobacco and cannabis dependence	88 (24.6)	24 (22.6)	32 (23.4)	32 (28.1)	
Electronic cigarette use					0.447
No	234 (65.5)	75 (70.8)	86 (62.8)	73 (64.0)	
Yes	84 (23.5)	18 (17.0)	37 (27.0)	29 (25.4)	
Yes, daily	39 (10.9)	13 (12.3)	14 (10.2)	12 (10.5)	
Any lifetime tobacco quit attempt	226 (63.3)	66 (62.3)	94 (68.6)	66 (57.9)	0.207
≥ 4 lifetime tobacco quit attempts	122 (34.2)	37 (34.9)	46 (33.6)	39 (34.2)	0.977
Any lifetime cannabis quit attempt	238 (66.7)	67 (63.2)	90 (65.7)	81 (71.1)	0.446
≥ 4 lifetime cannabis quit attempts	138 (38.7)	35 (33.0)	48 (35.0)	55 (48.2)	0.037
Any lifetime dual quit attempt	140 (39.2)	47 (44.3)	57 (41.6)	36 (31.6)	0.117
Would like to cut down one's tobacco consumption	259 (72.5)	73 (68.9)	105 (76.6)	81 (71.1)	0.368
Would like to cut down one's cannabis consumption	170 (47.6)	46 (43.4)	53 (38.7)	71 (62.3)	0.001
Currently receiving care for tobacco or cannabis use	41 (11.48)	12 (11.32)	11 (8.03)	18 (15.79)	0.158
According to you, based on your current patterns of use, which substance do you think poses the greatest risks to your health (physical or mental)?					0.002
Tobacco	177 (49.6)	44 (41.5)	83 (60.6)	50 (43.9)	
Cannabis	72 (20.2)	21 (19.8)	22 (16.1)	29 (25.4)	
Both equally	79 (22.1)	35 (33.0)	18 (13.1)	26 (22.8)	
I do not know	29 (8.1)	6 (5.7)	14 (10.2)	9 (7.9)	
Which substance do you perceive yourself as being most dependent on?					0.008
Tobacco	170 (47.6)	41 (38.7)	66 (48.2)	63 (55.3)	
Cannabis	98 (27.5)	33 (31.1)	40 (29.2)	25 (21.9)	
Both equally	54 (15.1)	14 (13.2)	18 (13.1)	22 (19.3)	
I am not dependent on either substance	35 (9.8)	18 (17.0)	13 (9.5)	4 (3.5)	
Not always mixing cannabis with tobacco when smoked	45 (12.6)	10 (9.4)	22 (16.1)	13 (11.4)	0.273
During a single occasion (e.g., a break, an evening), do you ever use tobacco without using cannabis?					0.034
Never or almost never	79 (22.13)	29 (27.36)	33 (24.09)	17 (14.91)	
Sometimes	162 (45.38)	48 (45.28)	66 (48.18)	48 (42.11)	
Always or almost always	116 (32.49)	29 (27.36)	38 (27.74)	49 (42.98)	
During a single occasion (e.g., a break, an evening), do you ever use cannabis without using tobacco?					0.069
Never or almost never	175 (49.02)	52 (49.06)	56 (40.88)	67 (58.77)	
Sometimes	129 (36.13)	40 (37.74)	55 (40.15)	34 (29.82)	
Always or almost always	53 (14.85)	14 (13.21)	26 (18.98)	13 (11.4)	
Do you know vaporizers?					0.243
No	149 (41.7)	51 (48.1)	54 (39.4)	44 (38.6)	
Yes, but no past‐month use	178 (49.9)	50 (47.2)	67 (48.9)	61 (53.5)	
Yes, past‐month use	30 (8.4)	5 (4.7)	16 (11.7)	9 (7.9)	

Abbreviations: DND, daily or near‐daily; IQR, interquartile range.

^a^ Chi‐square test for categorical variables and Kruskal‐Wallis test for numerical variables.

^b^ DND is understood as ≥ 20 days of use per month.

^c^ Tobacco dependence is defined as a Fagerström Test for Nicotine Dependence score ≥ 4 [44] and cannabis dependence is defined as a CUDIT‐Short Form score ≥ 2 [45].

### 3.2 Preference for Simultaneous Cessation (Binary Outcome)

In the multivariable binary logistic regression model, preferring simultaneous cessation (rather than quitting tobacco first or cannabis first) was positively associated with perceiving one's own tobacco and cannabis use equally risky for one's health (adjusted odds ratio 2.35, 95% confidence interval [1.30; 4.24], *p* = 0.005 vs. considering tobacco as riskier than cannabis), and perceiving oneself as more dependent on cannabis than on tobacco (2.01 [1.06; 3.80], *p* = 0.033) or perceiving oneself as not dependent on either substance (2.75 [1.23; 6.17], *p* = 0.014) as compared to perceiving oneself as more dependent on tobacco than cannabis. Lastly, preference for simultaneous cessation was inversely associated with high‐frequency use of both tobacco and cannabis (0.44 [0.21; 0.90], *p* = 0.024) compared with low‐frequency use of both substances (Table 2).

**TABLE 2:** Factors associated with preferring simultaneous cessation (logistic regression model, *n* = 357).

	aOR [95% CI]	*p*‐value
Gender		
Men	1	
Women	0.78 [0.47; 1.30]	0.344
Other/Do not want to answer	0.41 [0.08; 2.07]	0.283
Age, in years	1.04 [0.96; 1.11]	0.335
Frequency of use		
Non‐daily tobacco and non‐DND cannabis use	1	
Daily tobacco use, non‐DND cannabis use	0.97 [0.42; 2.22]	0.938
DND cannabis use, non‐daily tobacco use	0.56 [0.23; 1.39]	0.210
Daily tobacco and DND cannabis use	0.44 [0.21; 0.90]	0.024
According to you, based on your current patterns of use, which substance do you think poses the greatest risks to your health (physical or mental)?		
Tobacco	1	
Cannabis	1.16 [0.60; 2.24]	0.662
Both equally	2.35 [1.30; 4.24]	0.005
I do not know	0.63 [0.23; 1.73]	0.373
Which substance do you perceive yourself as being most dependent on?		
Tobacco	1	
Cannabis	2.01 [1.06; 3.80]	0.033
Both equally	1.24 [0.60; 2.58]	0.562
I am not dependent on either substance	2.75 [1.23; 6.17]	0.014
Not always mixing cannabis with tobacco when smoked	0.59 [0.26; 1.32]	0.198

Abbreviations: aOR, adjusted odds ratio; CI, confidence interval; DND, daily or near‐daily.

Results from the sensitivity analysis did not differ substantially from those of the main analysis (Supplementary Table 1).

In the alternative model including the dependence variable (but not the frequency variable), preference for simultaneous cessation was inversely associated with “tobacco dependence only” (0.24 [0.06; 0.95], *p* = 0.043), “cannabis dependence only” (0.38 [0.19; 0.77]), and almost significantly associated with “tobacco and cannabis dependence” (0.46 [0.21; 1.00], *p* = 0.051), as compared to “no dependence” (Supplementary Table 2).

### 3.3 Preference for Simultaneous Cessation (Three‐Level Outcome)

Results of the multinomial regression are provided in Supplementary Table 3. They did not differ substantially from those of the sensitivity analysis (Supplementary Table 4).

In the multivariable analysis, women had a higher probability of preferring cannabis cessation first than men (Supplementary Figure 1).

**FIGURE 1:**
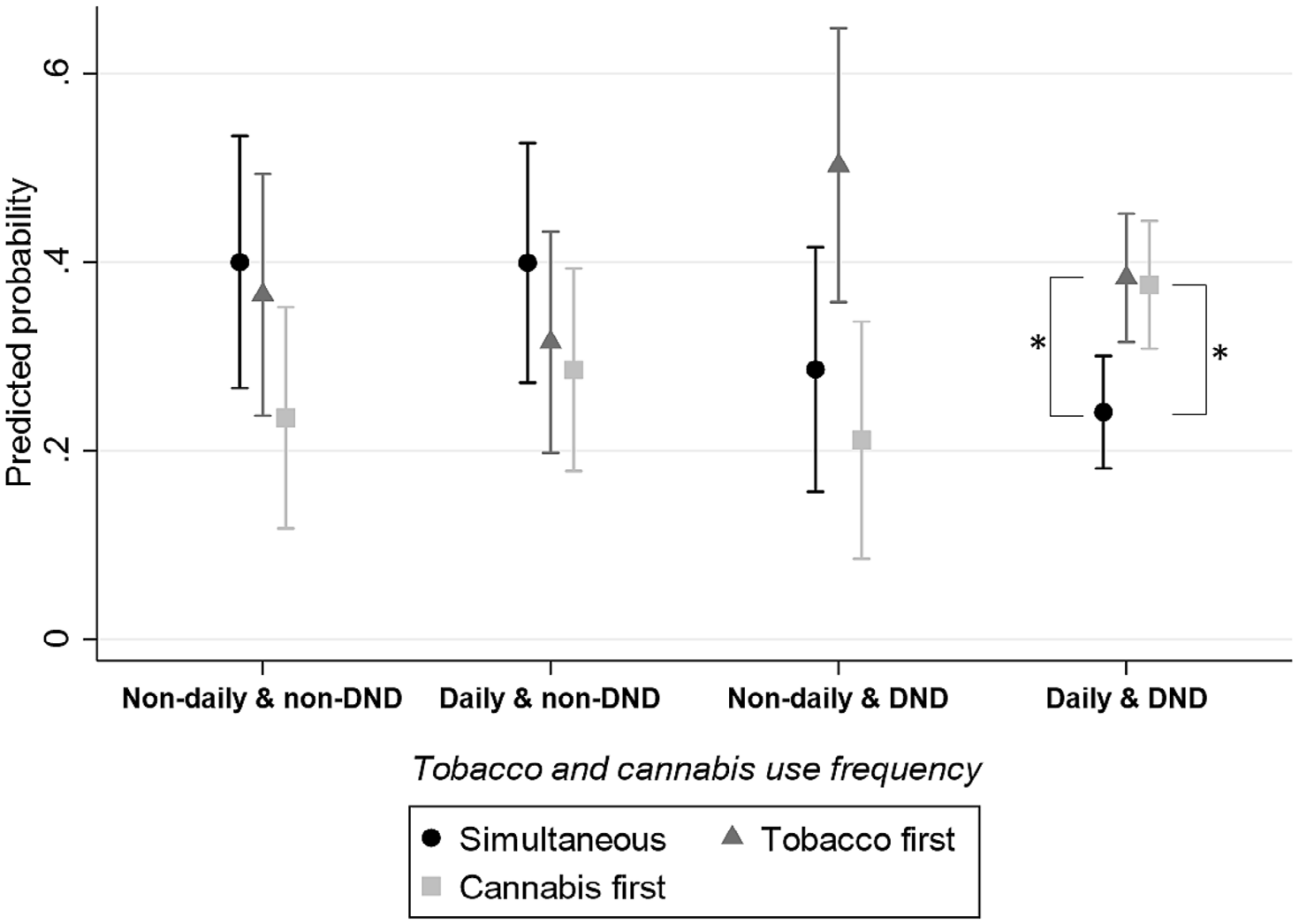
Predicted probabilities of preferring simultaneous or sequential cessation according to tobacco and cannabis use frequency (multinomial regression model, *n* = 357). DND, daily or near‐daily use. Daily and non‐daily refer to tobacco use. DND and non‐DND refer to cannabis use. *: *P* < 0.05, pairwise comparisons of predicted probabilities (Wald test), adjusted with Bonferroni correction.

Participants with a high frequency of use of both substances had a lower probability of preferring simultaneous cessation than the other two options. Those who used tobacco non‐daily but cannabis daily or near‐daily had a close‐to‐significant higher probability of preferring tobacco cessation first than cannabis cessation first (*p* = 0.050) (Figure 1).

**FIGURE 2:**
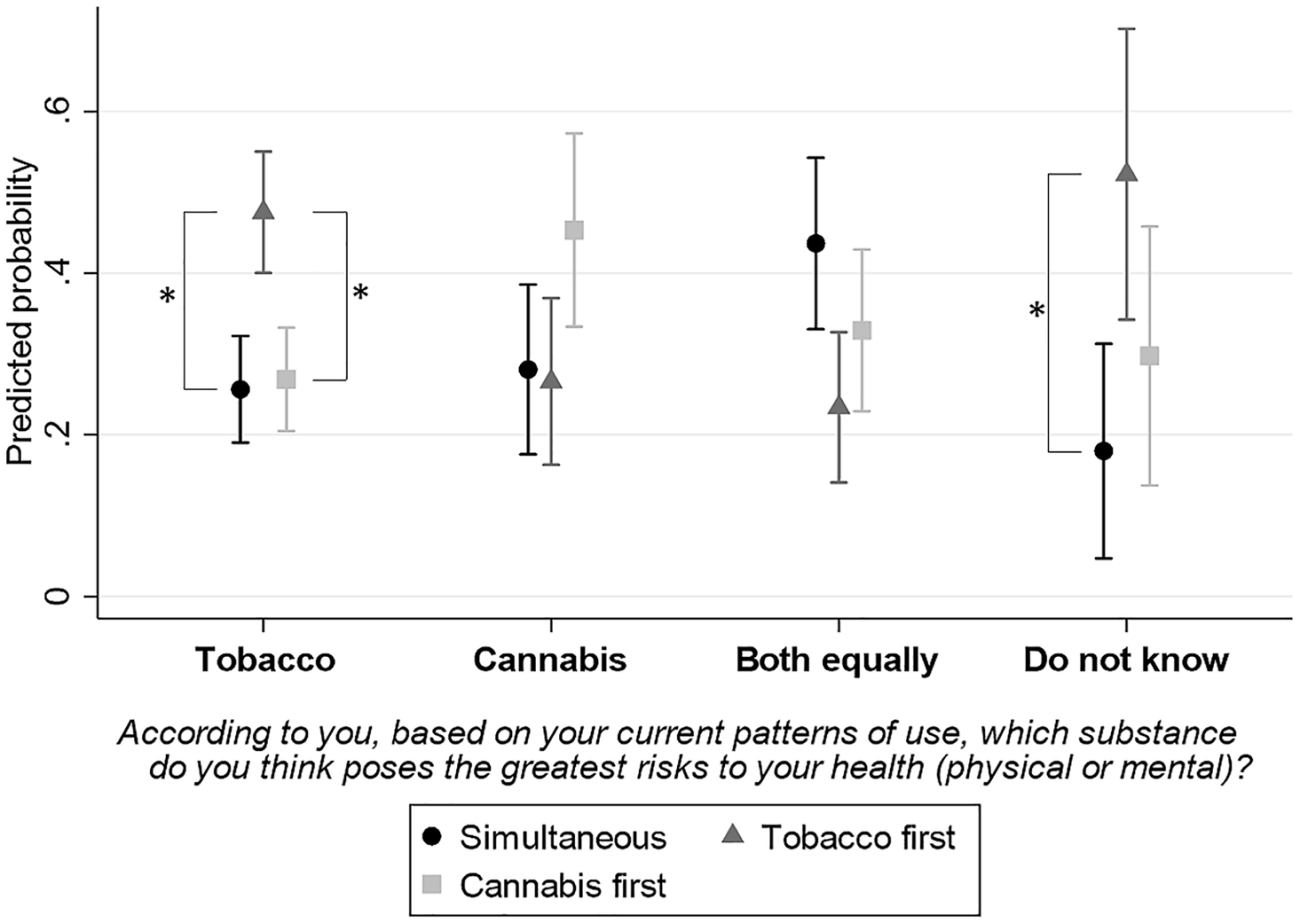
Predicted probabilities of preferring simultaneous or sequential cessation according to relative risk perception of one's own pattern of use (multinomial regression model, *n* = 357). *: *p* < 0.05, pairwise comparisons of predicted probabilities (Wald test), adjusted with Bonferroni correction.

Among participants who considered their use of both substances equally risky for their health, the probability of preferring simultaneous cessation was higher than among those who considered their tobacco use riskier and those who did not know which use was riskier for themselves (Figure 2). In participants who considered their tobacco use riskier, the probability of preferring tobacco cessation first was higher than in those who perceived their cannabis use as riskier and those who considered their two uses equally risky. Among participants who did not know which use was riskier, the probability of preferring tobacco cessation first was higher than the one in those who perceived their two uses as equally risky.

In participants who considered both uses as equally risky, the probability of preferring simultaneous cessation was close‐to‐significantly higher than that of preferring tobacco cessation first (*p* = 0.063, Figure 2).

**FIGURE 3:**
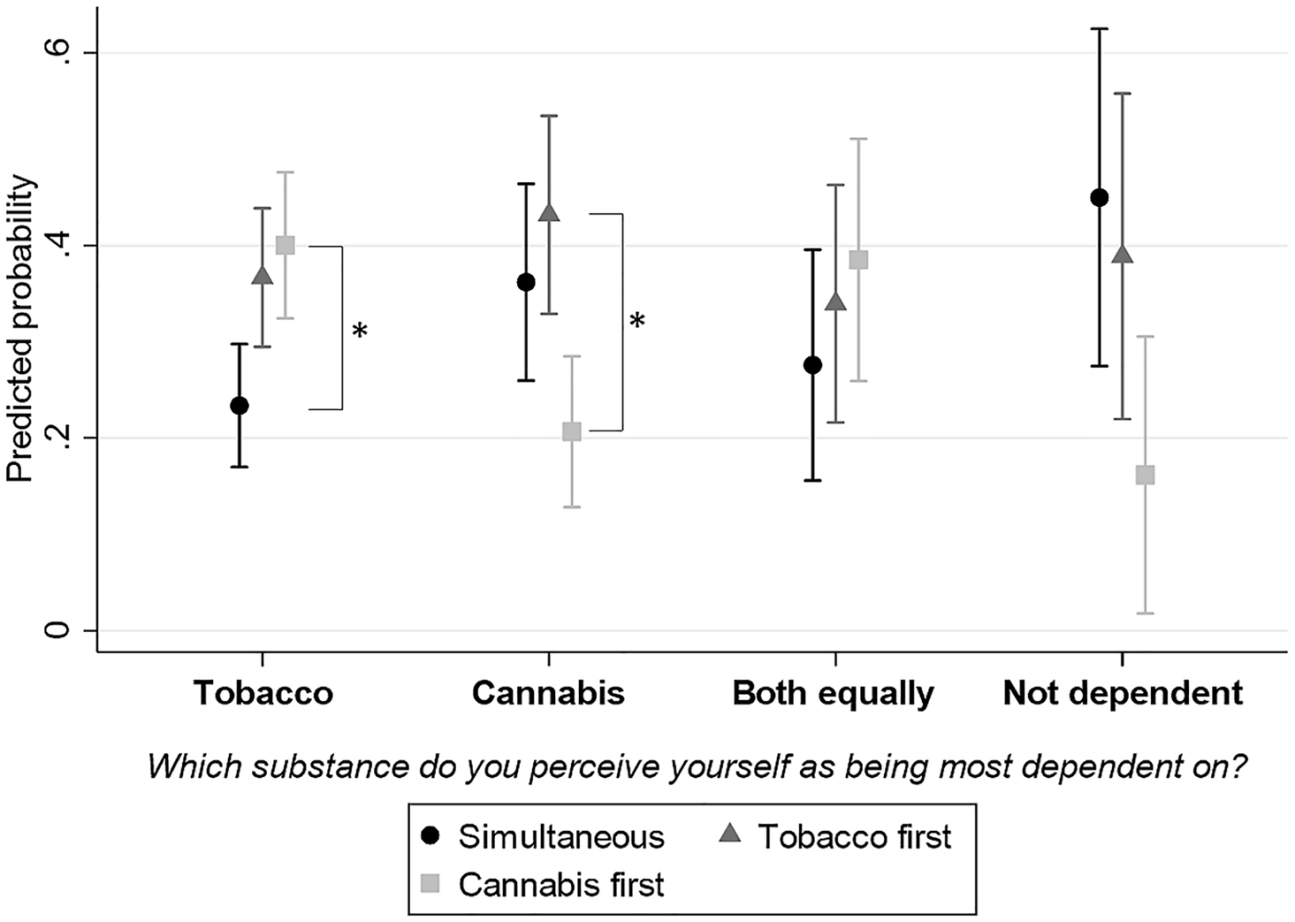
Predicted probabilities of preferring simultaneous or sequential cessation according to relative perception of dependence (multinomial regression model, *n* = 357). *: *p* < 0.05, pairwise comparisons of predicted probabilities (Wald test), adjusted with Bonferroni correction.

In participants who considered themselves more dependent on tobacco than on cannabis, the probability of preferring cannabis cessation first was higher than that in those who considered themselves more dependent on cannabis, and higher than that in those who did not consider themselves dependent on either substance (Figure 3).

Mixing or not mixing cannabis with tobacco was not associated with any preference (Supplementary Figure 2).

In the alternative model including the dependence variable (but not the frequency variable), participants reporting no dependence had a higher probability of preferring simultaneous cessation than preferring cannabis cessation first. Those who were dependent on cannabis but not on tobacco had a higher probability of preferring tobacco cessation first than simultaneous cessation (Supplementary Figure 3).

### 3.4 Preferred Types of Quitting Methods During Previous Cessation Attempts

Among participants who had ever attempted to quit tobacco (*n* = 226), the most reported methods were electronic cigarette (52.7%), sport (26.5%), and nicotine replacement therapy (24.8%). More than one in five participants (21.7%) reported using none of the listed methods (Supplementary Figure 4). Those who used electronic cigarettes were more likely to report electronic cigarettes (70.3%) than the remaining ones (40.7%, *p* < 0.001).

Among those who had ever attempted to quit cannabis (*n* = 238), the most reported methods were cannabidiol (CBD) (44.1%), none (29.0%), electronic cigarette (26.1%) and sport (24.8%) (Supplementary Figure 4). Those who used electronic cigarettes were more likely to report electronic cigarettes (39.7%) than the remaining ones (17.2%, *p* < 0.001).

### 3.5 Preferred Types of Quitting Methods for Future Cessation Attempts

**FIGURE 4:**
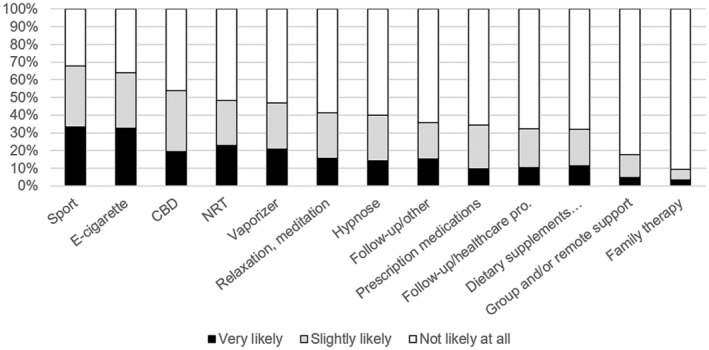
Likelihood of using predefined types of methods in case of tobacco cessation in the coming month (*n* = 357). CBD, cannabidiol; NRT, nicotine replacement therapy.

**FIGURE 5:**
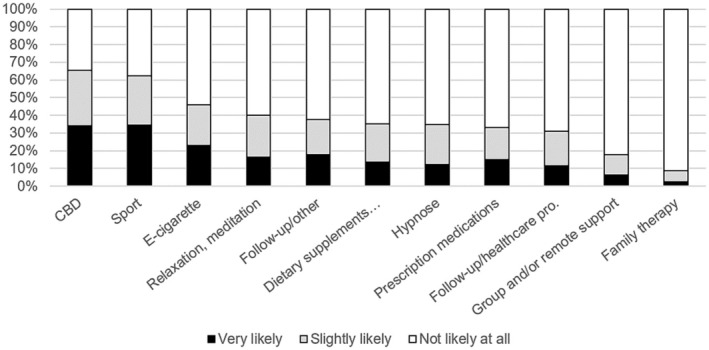
Likelihood of using predefined types of methods in case of cannabis cessation in the coming month (*n* = 357). CBD, cannabidiol.

For tobacco cessation, sport (33.3% “very likely”) and electronic cigarette (32.8% “very likely”) were the most preferred types of methods (< 36.2% “not at all”) (Figure 4). Those who used electronic cigarettes were more likely to report “slightly likely” or “very likely” for it (90.2%) than the remaining ones (50.0%, *p* < 0.001). For cannabis cessation, CBD (34.2% “very likely”) and sport (34.5% “very likely”) were the most preferred types of methods (< 37.9% “not at all”) (Figure 5). Those who used electronic cigarettes were more likely to report “slightly likely” or “very likely” for it (68.3%) than the remaining ones (34.2%, *p* < 0.001).

For the methods that were applicable to both substances, *ρ* was ≥ 0.73 (CBD) and ≤ 0.94 (family therapy), and all Bonferroni‐adjusted *p*‐values were < 0.001.

These likelihoods varied according to whether participants preferred simultaneous or sequential cessation (Supplementary Table 5). For tobacco cessation, participants preferring simultaneous cessation were less likely to use nicotine replacement therapy, CBD, electronic cigarette (than those preferring cannabis cessation first) and a vaporizer (than those preferring tobacco cessation first). For cannabis cessation, they were less likely to use CBD and less likely to use an electronic cigarette (than those preferring cannabis cessation first).

Participants who preferred simultaneous cessation declared a median [interquartile range] number of 3.5 [1; 7] types of methods they would likely or very likely try for next‐month tobacco cessation attempts, which was lower than those who preferred tobacco (5 [3; 7], Kruskal‐Wallis test *p* = 0.022) or cannabis (5 [3; 7], Kruskal‐Wallis test *p* = 0.005) cessation first. For next‐month cannabis cessation, it was 2 [0; 5], which was lower than 3 [1; 6] (tobacco first, *p* = 0.035), and 3 [1; 5] (cannabis first, *p* = 0.059).

For tobacco cessation, 6.4% of the study sample reported that they would not be likely to use any of the proposed methods, and they were 17.9% for cannabis cessation (exact McNemar significance probability < 0.001). For future tobacco cessation attempts, the percentages of participants who reported none of the proposed methods were 14.2%, 4.4% and 1.8% for those who preferred simultaneous, tobacco first and cannabis first, respectively (chi‐square test *p* < 0.001). For cannabis cessation, it was 30.2%, 13.9% and 11.4% (*p* < 0.001).

## 4 Discussion

In a gender‐balanced sample of young adults co‐using tobacco and cannabis in France, we observed overall, comparable proportions of preference for the three dual cessation strategies: simultaneous cessation, quitting tobacco first and quitting cannabis first. However, preference for simultaneous cessation appeared to be more frequently reported among people with a less frequent or non‐dependent pattern of co‐use. Electronic cigarette, CBD and sport (plus nicotine replacement therapy for tobacco) were the most cited types of methods previously used and likely to be used for future quit attempts for both substances.

Simultaneous cessation, while generally considered acceptable in previous studies [34], is therefore not an obvious choice when young adults who co‐use have to consider quitting both substances. After multiple adjustments, we found that preference for simultaneous cessation was less probable than other choices in people with frequent use of both substances or, alternatively, in people with tobacco or cannabis dependence according to validated scales. It was also, to some extent, a more probable choice in people who perceived themselves, or were identified, as non‐dependent on either substance. Therefore, simultaneous cessation seems to be preferred by people engaged in lower‐frequency or non‐dependent co‐use, but not for people most at risk of co‐use‐related harms. Those results also imply that simultaneous cessation is preferred by people who may face less difficulty in quitting tobacco and cannabis. Indeed, it has been observed that for both substances, the higher the consumption level, the poorer the cessation outcomes [70, 71, 72, 73].

People who felt more dependent on one substance tended to prefer to quit the other first—a strategy of “starting with the easier one.” Although continuing one substance can trigger relapse to the other, quitting one may also increase the likelihood of successfully stopping the second [74, 75], supporting this “easier first” strategy.

First, tobacco cessation outcomes are poorer among people who co‐use cannabis [26, 27, 28, 29], meaning that successful cannabis cessation first may increase the likelihood of quitting tobacco in a second time, despite a transient increase in tobacco smoking after cannabis cessation [76]. Regarding tobacco cessation first, a 2017 review concluded that tobacco smoking cessation does not appear to have a negative effect and often has a positive effect on substance use outcomes [77]. Specifically, two studies previously found that tobacco cessation had positive effects on cannabis use frequency in people with heavy alcohol use [78], and on cannabis relapse in adolescents seeking treatment for substance use [79]. While poorly documented, we may lastly expect that a first success in substance cessation may increase self‐esteem, self‐worth, self‐confidence, or self‐efficacy, that will in turn improve the chances of a second successful cessation [80, 81].

We observed a tendency for participants to prefer quitting first the substance perceived as more harmful. Health concerns are common motives for quitting tobacco and cannabis [82, 83], and tobacco is generally perceived as more harmful than cannabis by young people [84, 85]. Our findings suggest that, for individuals not ready for simultaneous cessation, providing clear and complementary information on health risks may support a sequential approach starting with the substance perceived as more harmful.

Our results therefore pointed out the fact that sequential cessation may be preferred and more suited to some groups of people who co‐use. Respecting patients' preferences generally improves substance use treatment outcomes [86, 87]. Therefore, there is a need to explore how sequential cessation could be feasible, and how it may be prepared, accompanied and improved. For instance, mulling (i.e., mixing tobacco with cannabis within joints), which is common in Europe and France [19, 88], may be a barrier to tobacco cessation first. Decoupling cannabis use from its mixture with tobacco may therefore be necessary prior to implementing such a sequential cessation. One way to do so may be to switch from joints to vaporizers for cannabis administration [89, 90]. While expensive and probably not adapted for some people who co‐use, such a cessation method seems acceptable by some, according to our results on methods likely to be used. Moreover, progressive replacement of tetrahydrocannabinol (THC)‐rich cannabis with tetrahydrocannabinol‐low cannabis (generally rich in CBD) within joints to reach tobacco‐containing but THC‐low or THC‐free joints deserves further investigation [53, 91], as CBD appeared highly acceptable in our study sample.

The interrelatedness of tobacco and cannabis use has been reported related to patterns of use [92]. Future studies are needed to further explore how temporal and contextual proximity between tobacco and cannabis use influences preferences for sequential versus simultaneous quitting, as well as the success of these cessation strategies. In our sample, few participants reported smoking “pure” cannabis (i.e., in tobacco‐free joints). Consequently, our analyses yielded wide confidence intervals and the preference for quitting tobacco first—compared to simultaneous cessation—was not significantly associated with “not always mixing,” despite a relatively high adjusted odds ratio. Regarding same‐occasion use patterns, our descriptive analyses suggest that participants who preferred quitting cannabis first were more likely to commonly use tobacco without using cannabis during a single occasion. Inversely, those who preferred to quit tobacco first were more likely to commonly use cannabis without using tobacco during a single occasion (though this pattern was infrequent). These findings suggest that complementary use (i.e., when one substance enhances or completes the effects of the other) may act as a barrier to sequential cessation. In contrast, independent use of each substance may facilitate the success of sequential quitting strategies. One of the mechanisms that could explain these patterns is the triggering of both substances' use by shared conditioned cues, acquired through their concurrent use, as well as the triggering of one substance's use by the use of the other [93]. Taken together, these findings highlight the need for more precise data collection on patterns of co‐use in future research, particularly regarding same‐occasion use and co‐administration of tobacco and cannabis.

While it is acknowledged that both substances should be taken into account in case of co‐use treatment [94], to date, there are no clinical practice guidelines for treating tobacco‐cannabis co‐use [34]. Regarding tobacco cessation in young people, a 2017 Cochrane review concluded that evidence remains limited for all intervention types, whether they are behavioural or pharmacological [95]. For CUD treatment generally, evidence is also limited regarding the effectiveness of psychological and pharmacological interventions [96, 97]. For tobacco cessation, the French National Authority for Health recommended in 2023 care by a healthcare professional to ensure follow‐up, as well as the initiation of pharmacological treatment if necessary (nicotine replacement therapy being the first‐line treatment). Vaping products “could be used for specific smokers and/or vulnerable populations” (such as people with co‐addictions), but “only in the event of failure or poor adherence to treatment, and when the target populations explicitly express a preference for vaping devices”, and with tobacco cessation as a target [49]. There is no approved treatment, nor official published treatment guidelines for CUD in France.

In our sample, 34.5% of participants used electronic cigarette (10.9% for daily use, a figure close to the one observed in adults who co‐use tobacco and cannabis in France in 2021 (8.4%) [16]). Among these users, almost two‐thirds reported using it to reduce or quit tobacco. This is consistent with what was reported among dual U.S. adults who use cigarettes and electronic cigarettes [98]. Accordingly, in our sample, most participants who used electronic cigarette had previously attempted to quit tobacco using it, and most also intended to use it in future tobacco cessation attempts. Such a dual (combustible and electronic) cigarette use is common. It may be an interim state for people attempting to quit cigarette smoking, while its impact on smoking cessation is uncertain [99].

With sport, CBD and electronic cigarette as the methods most likely to be used for both tobacco and cannabis cessation, our results suggest, as Walsh et al. in the UK [35], that recourse to formal care and evidence‐based interventions is low among French young adults who co‐use. However, we found that around 50% of our sample were somehow likely to use nicotine replacement therapy for tobacco cessation. In contrast, the likelihood of being followed up by a health professional was very low for both substances.

Contrary to nicotine‐containing e‐cigarettes, which are effective for tobacco cessation [100, 101, 102], no consistent evidence supports the effectiveness of exercise interventions for tobacco cessation [103]. Evidence for CBD in reducing or quitting tobacco and cannabis use also remains limited [104]. Nevertheless, sport, CBD and vaping appear to be preferred by young adults and warrant further investigation as potential treatments for co‐use.

Existing evidence regarding these methods has rarely been drawn from the relevant population in terms of age and type of use. Although few studies have explored exercise for substance use treatment in youth [105], physical activity is expected to alleviate anxiety and depression and improve cognitive function among individuals with substance use disorders [106]. These benefits are particularly relevant given the burden of mental health conditions among European young people [107], which are strongly associated with both tobacco and cannabis use, as well as co‐use [16, 108, 109]. Emerging data also suggest that physical activity may be linked to lower risks of CUD [110, 111], and provide both behavioural and physiological rationales for further research [112]. Evidence supporting oral CBD for reducing cannabis or tobacco/nicotine use remains weak [113, 114, 115, 116], although vaping CBD may hold promise for cannabis use reduction [117].

There is therefore promising research perspective in testing and combining physical exercise, electronic cigarette and CBD (potentially with other types of interventions) to treat tobacco and cannabis co‐use in young adults. Indeed, each method presents rationale for such use, appeared acceptable for both substances, and could therefore be applied as a common strategy to quit tobacco and cannabis, either simultaneously or sequentially. Indeed, we have shown that, for a given method, the likelihoods of using it for both substances were correlated.

When looking at types of methods according to preferred cessation approach, we observed that participants preferring simultaneous cessation declared less methods they would be likely to use for cessation, and that they were more likely to declare none. This is consistent with the previously discussed interpretation of the results, suggesting that simultaneous cessation may be preferred by individuals with a lower‐risk profile of use, who therefore perceive less need for external resources to quit.

Between 20% and 30% of participants who had previously tried to quit (depending on the substance) reported using none of the listed cessation methods. High rates of unassisted tobacco quit attempts among young adults who co‐use were observed in the UK, where most still expected to seek support from their family doctor [35]. A qualitative study among young adults who co‐use electronic and combustion cigarettes in California also reported that “cold turkey” quitting was frequently tried for quitting either or both tobacco products [118]. In the US, young adults may also be less likely than older adults to use pharmacotherapy [57].

In France, qualitative studies are needed to understand why unassisted cessation is so likely among young adults who co‐use. Possible reasons include limited awareness of recommended methods, negative beliefs about treatments [119], barriers to care [120], preference for self‐reliance [121, 122] and cannabis‐related stigma [121, 122, 123]. Consistently, the cessation methods most often considered in our sample reflected this autonomy: professional or group support was rarely mentioned, while preferred strategies—such as alternative products or behaviours—could be implemented independently.

The major strength of the present study is its originality, as to our knowledge, preference for simultaneous versus sequential dual cessation had not been explored among people who co‐use so far. Those findings were extended by providing, for the same individuals, preferences for cessation strategies for both substances. Our results may therefore directly inform clinicians and also pave the way for future research on co‐use treatment.

Despite its nationwide implementation and gender balance, generalisability of our findings should be taken with caution, particularly given that the questionnaire was self‐administered, age was not verified, and no attention checks or bot detection mechanisms were applied. Our study sample was a convenient one and its modest size may have prevented some effects from being identified (e.g., non‐mixing participants were very few). The predefined list of quitting methods may have lacked exhaustiveness. For instance, technology‐based programmes may have warranted separate, dedicated items [124]. Additionally, some methods’ labels may have been interpreted differently by participants. For example, the term “sport” did not presuppose specific ways of appropriating physical activity—whether as a distraction, a source of motivation, or a means of managing withdrawal. Moreover, most participants were non‐treatment‐seeking and our results require replication in clinical samples.

To conclude, there was no overarching preference for simultaneous or sequential dual cessation in our sample of young adults who co‐used tobacco and cannabis. However, simultaneous cessation seemed preferred by participants with a low‐risk use profile, suggesting that feasible and effective sequential strategies need to be developed for people most in need for treatment. Future research should include acceptable strategies such as physical exercise, CBD and electronic cigarettes to identify effective means to accompany dual cessation in young adults.

## Author Contributions

T.B.: conceptualization, formal analysis, funding acquisition, investigation, project administration, writing – original draft preparation, writing – review and editing. G.C.: investigation, project administration, writing – review and editing. V.L.: data curation, writing – review and editing. G.M.: investigation. C.C.: investigation, writing – review and editing. J.T.: Investigation, writing – review and editing. C.M.: investigation, writing – review and editing. P.C.: funding acquisition, writing – review and editing.

## Funding

This study received funding from the 2023 call for projects on ‘Psychoactive substances and addictive behaviours’ (AAP‐2023‐SPA‐17, project #23IReSP093), managed by the IReSP (French Institute for Public Health Research) and the INCa (French National Cancer Institute).

## Conflicts of Interest

The authors declare no conflicts of interest.

## Supporting information


**Supplementary Figure 1:** Predicted probabilities of preferring simultaneous or sequential cessation according to gender (multinomial regression model, *n* = 357).

**Supplementary Figure 2:** Predicted probabilities of preferring simultaneous or sequential cessation according to mixing cannabis with tobacco when smoking cannabis (multinomial regression model, *n* = 357).

**Supplementary Figure 3:** Predicted probabilities of preferring simultaneous or sequential cessation according to tobacco and cannabis dependence (multinomial regression model, *n* = 357).

**Supplementary Figure 4:** Types of quitting methods tried during previous tobacco (*n* = 226) and cannabis (*n* = 238) cessation attempts.

**Supplementary Table 1:** Factors associated with preference for simultaneous dual cessation (sensitivity analysis, logistic regression, *n* = 316).

**Supplementary Table 2:** Factors associated with preferring simultaneous cessation (alternative logistic regression model, *n* = 357).

**Supplementary Table 3:** Factors associated with preference for the type of dual cessation (multinomial regression, *n* = 357).

**Supplementary Table 4:** Factors associated with preference for the type of dual cessation (multinomial regression, sensitivity analysis, *n* = 316).

**Supplementary Table 5:** Participants' preferred types of quitting methods according to preference for simultaneous or sequential cessation.

## Data Availability

The data that support the findings of this study are available on request from the corresponding author. The data are not publicly available due to privacy or ethical restrictions.
