# Supplementary material for: Simultaneous versus Sequential Cessation of Tobacco and Cannabis: Preferences of Young Adults Who Co‐Use in France
Source: Drug Alcohol Rev. 2026 Aug 9;45(6):e70221. doi: 10.1111/dar.70221 (PMC13453397; doi:10.1111/dar.70221)
Supplement: Supplementary file 2 [file DAR-45-0-s002.docx]

**Supplementary Table 5. Participants’ preferred types of quitting methods according to preference for simultaneous or sequential cessation**

|  |  | **Total** | **Simultaneous** | **Tobacco first** | **Cannabis first** |  |
| --- | --- | --- | --- | --- | --- | --- |
|  |  | **N (%)** | **N (%)** | **N (%)** | **N (%)** | **p-value^1^** |
| **If you were to quit tobacco in the coming month, would you be likely to...** | Use nicotine replacement therapy (any form: patch, gum, etc.)? | 173 (48.5) | 39 (36.8)^ab^ | 73 (53.3)^a^ | 61 (53.5)^b^ | 0.016 |
|  | Use prescription medications? | 123 (34.5) | 35 (33.0) | 52 (38.0) | 36 (31.6) | 0.533 |
|  | Use dietary supplements, essential oils, homeopathy, or over-the-counter medications? | 114 (31.9) | 31 (29.2) | 39 (28.5) | 44 (38.6) | 0.179 |
|  | Use electronic cigarettes? | 228 (63.9) | 57 (53.8)^a^ | 85 (62.0) | 86 (75.4)^a^ | 0.003 |
|  | Use CBD (oil, capsules, etc.)? | 193 (54.1) | 44 (41.5)^ab^ | 81 (59.1)^a^ | 68 (59.6)^b^ | 0.008 |
|  | Turn to follow-up with a healthcare professional (physician, midwife, nurse, physiotherapist, dentist, pharmacist, etc.)? | 115 (32.2) | 32 (30.2) | 42 (30.7) | 41 (36.0) | 0.581 |
|  | Turn to follow-up with another professional (coach, psychologist, etc.)? | 128 (35.9) | 35 (33.0) | 49 (35.8) | 44 (38.6) | 0.689 |
|  | Use group and/or remote support (telephone, online, SMS, group meetings, etc.)? | 63 (17.6) | 17 (16.0) | 28 (20.4) | 18 (15.8) | 0.551 |
|  | Use relaxation/meditation or related approaches? | 148 (41.5) | 40 (37.7) | 58 (42.3) | 50 (43.9) | 0.631 |
|  | Use hypnosis? | 143 (40.1) | 39 (36.8) | 59 (43.1) | 45 (39.5) | 0.606 |
|  | Use sports? | 242 (67.8) | 68 (64.2) | 92 (67.2) | 82 (71.9) | 0.458 |
|  | Use a vaporizer (to decouple cannabis use from tobacco use)? | 168 (47.1) | 39 (36.8)^a^ | 75 (54.7)^a^ | 54 (47.4) | 0.021 |
|  | Use family therapy? | 34 (9.5) | 7 (6.6) | 17 (12.4) | 10 (8.8) | 0.294 |
| **If you were to quit cannabis in the coming month, would you be likely to…** | Use prescription medications? | 118 (33.1) | 34 (32.1) | 49 (35.8) | 35 (30.7) | 0.675 |
|  | Use dietary supplements, essential oils, homeopathy, or over-the-counter medications? | 126 (35.3) | 32 (30.2) | 51 (37.2) | 43 (37.7) | 0.422 |
|  | Use electronic cigarettes? | 164 (45.9) | 39 (36.8)^a^ | 62 (45.3) | 63 (55.3)^a^ | 0.023 |
|  | Use CBD (oil, capsules, etc.)? | 233 (65.3) | 55 (51.9)^ab^ | 98 (71.5)^a^ | 80 (70.2)^b^ | 0.003 |
|  | Turn to follow-up with a healthcare professional (physician, midwife, nurse, physiotherapist, dentist, pharmacist, etc.)? | 111 (31.1) | 30 (28.3) | 46 (33.6) | 35 (30.7) | 0.674 |
|  | Turn to follow-up with another professional (coach, psychologist, etc.)? | 134 (37.5) | 34 (32.1) | 57 (41.6) | 43 (37.7) | 0.314 |
|  | Use group and/or remote support (telephone, online, SMS, group meetings, etc.)? | 64 (17.9) | 18 (17.0) | 31 (22.6) | 15 (13.2) | 0.143 |
|  | Use relaxation/meditation or related approaches? | 143 (40.1) | 44 (41.5) | 54 (39.4) | 45 (39.5) | 0.936 |
|  | Use hypnosis? | 124 (34.7) | 34 (32.1) | 51 (37.2) | 39 (34.2) | 0.698 |
|  | Use sports? | 222 (62.2) | 66 (62.3) | 85 (62.0) | 71 (62.3) | 0.999 |
|  | Use family therapy? | 31 (8.7) | 8 (7.5) | 15 (10.9) | 8 (7.0) | 0.482 |

**^1^** Chi-square test

Common superscript letters indicate significant differences between the groups (paired chi-square tests adjusted for Bonferroni correction, p<0.005).
