# Supplementary material for: Simultaneous versus Sequential Cessation of Tobacco and Cannabis: Preferences of Young Adults Who Co‐Use in France
Source: Drug Alcohol Rev. 2026 Aug 9;45(6):e70221. doi: 10.1111/dar.70221 (PMC13453397; doi:10.1111/dar.70221)
Supplement: Supplementary file 3 [file DAR-45-0-s003.docx]

**Supplementary Table 3. Factors associated with preference for the type of dual cessation (multinomial regression, n=357)**

|  | **Tobacco first vs. simultaneous** |  | **Cannabis first vs. simultaneous** |  | **Cannabis first vs. tobacco first** |  |
| --- | --- | --- | --- | --- | --- | --- |
|  | **aRRR** | **p-value** | **aRRR** | **p-value** | **aRRR** | **p-value** |
| **Gender** |  |  |  |  |  |  |
| Men (ref.) | 1 |  |  |  |  |  |
| Women | 0.93 [0.52;1.65] | 0.800 | 1.77 [0.98;3.19] | 0.058 | 1.91 [1.09;3.34] | 0.024 |
| Other / Do not want to answer | 3.46 [0.68;17.62] | 0.135 | 1.47 [0.23;9.53] | 0.687 | 0.42 [0.11;1.71] | 0.228 |
| **Age**, in years | 0.95 [0.88;1.03] | 0.237 | 0.99 [0.90;1.08] | 0.768 | 1.04 [0.96;1.13] | 0.381 |
| **Frequency of use** |  |  |  |  |  |  |
| Non-daily tobacco and non-DND cannabis use (ref.) | 1 |  | 1 |  | 1 |  |
| Daily tobacco use, non-DND cannabis use | 0.85 [0.33;2.23] | 0.745 | 1.23 [0.44;3.43] | 0.687 | 1.45 [0.52;4.03] | 0.479 |
| DND cannabis use, non-daily tobacco use | 2.05 [0.76;5.53] | 0.156 | 1.29 [0.38;4.30] | 0.683 | 0.63 [0.20;1.98] | 0.426 |
| Daily tobacco and DND cannabis use | 1.83 [0.82;4.10] | 0.143 | 2.88 [1.17;7.05] | 0.021 | 1.57 [0.66;3.74] | 0.305 |
| **According to you, based on your current patterns of use, which substance do you think poses the greatest risks to your health (physical or mental)?** |  |  |  |  |  |  |
| Tobacco (ref.) | 1 |  | 1 |  | 1 |  |
| Cannabis | 0.50 [0.22;1.11] | 0.089 | 1.65 [0.73;3.71] | 0.228 | 3.30 [1.51;7.21] | 0.003 |
| Both equally | 0.27 [0.13;0.55] | <0.001 | 0.71 [0.35;1.43] | 0.336 | 2.60 [1.24;5.46] | 0.011 |
| I do not know | 1.60 [0.54;4.74] | 0.398 | 1.63 [0.49;5.35] | 0.424 | 1.02 [0.38;2.70] | 0.973 |
| **Which substance do you perceive yourself as being most dependent on?** |  |  |  |  |  |  |
| Tobacco (ref.) | 1 |  | 1 |  | 1 |  |
| Cannabis | 0.76 [0.37;1.56] | 0.450 | 0.31 [0.14;0.68] | 0.003 | 0.41 [0.20;0.86] | 0.018 |
| Both equally | 0.77 [0.32;1.83] | 0.553 | 0.80 [0.35;1.86] | 0.610 | 1.05 [0.48;2.28] | 0.911 |
| I am not dependent on either substance | 0.53 [0.21;1.35] | 0.183 | 0.19 [0.05;0.66] | 0.009 | 0.36 [0.10;1.26] | 0.109 |
| **Not always mixing cannabis with tobacco when smoked** | 1.84 [0.78;4.30] | 0.161 | 1.50 [0.58;3.83] | 0.401 | 0.81 [0.37;1.80] | 0.613 |

aRRR, adjusted relative risk ratio; DND, daily or near-daily
