# Supplementary material for: Simultaneous versus Sequential Cessation of Tobacco and Cannabis: Preferences of Young Adults Who Co‐Use in France
Source: Drug Alcohol Rev. 2026 Aug 9;45(6):e70221. doi: 10.1111/dar.70221 (PMC13453397; doi:10.1111/dar.70221)
Supplement: Supplementary file 4 [file DAR-45-0-s004.docx]

**Supplementary Figure 3. Predicted probabilities of preferring simultaneous or sequential cessation according to tobacco and cannabis dependence (multinomial regression model, n=357)**

Tobacco dependence is defined as a Fagerström Test for Nicotine Dependence score ≥ 4 (39), and cannabis dependence is defined as a CUDIT-Short Form score ≥ 2 (40)
