# Supplementary material for: Simultaneous versus Sequential Cessation of Tobacco and Cannabis: Preferences of Young Adults Who Co‐Use in France
Source: Drug Alcohol Rev. 2026 Aug 9;45(6):e70221. doi: 10.1111/dar.70221 (PMC13453397; doi:10.1111/dar.70221)
Supplement: Supplementary file 5 [file DAR-45-0-s005.docx]

**Supplementary Figure 1. Predicted probabilities of preferring simultaneous or sequential cessation according to gender (multinomial regression model, n=357)**
