# Supplementary material for: Simultaneous versus Sequential Cessation of Tobacco and Cannabis: Preferences of Young Adults Who Co‐Use in France
Source: Drug Alcohol Rev. 2026 Aug 9;45(6):e70221. doi: 10.1111/dar.70221 (PMC13453397; doi:10.1111/dar.70221)
Supplement: Supplementary file 6 [file DAR-45-0-s006.docx]

**Supplementary Figure 4**. **Types of quitting methods tried during previous tobacco (n=226) and cannabis (n=238) cessation attempts**

Exact wording of the types of methods are provided in **Supplementary Table 4**.
