# Supplementary material for: Simultaneous versus Sequential Cessation of Tobacco and Cannabis: Preferences of Young Adults Who Co‐Use in France
Source: Drug Alcohol Rev. 2026 Aug 9;45(6):e70221. doi: 10.1111/dar.70221 (PMC13453397; doi:10.1111/dar.70221)
Supplement: Supplementary file 7 [file DAR-45-0-s007.docx]

**Supplementary Table 1. Factors associated with preference for simultaneous dual cessation (sensitivity analysis, logistic regression, n=316)**

|  | **aOR [95% CI]** | **p-value** |
| --- | --- | --- |
| **Gender** |  |  |
| Men | 1 |  |
| Women | 0.63 [0.37;1.09] | 0.099 |
| Other / Do not want to answer | 0.21 [0.02;1.92] | 0.166 |
| **Age**, in years | 1.03 [0.95;1.12] | 0.427 |
| **Frequency of use** |  |  |
| Non-daily tobacco and non-DND cannabis use | 1 |  |
| Daily tobacco use, non-DND cannabis use | 1.00 [0.43;2.36] | 0.995 |
| DND cannabis use, non-daily tobacco use | 0.43 [0.16;1.16] | 0.095 |
| Daily tobacco and DND cannabis use | 0.41 [0.19;0.86] | 0.019 |
| **According to you, based on your current patterns of use, which substance do you think poses the greatest risks to your health (physical or mental)?** |  |  |
| Tobacco | 1 |  |
| Cannabis | 0.98 [0.47;2.03] | 0.953 |
| Both equally | 2.51 [1.34;4.72] | 0.004 |
| I do not know | 0.66 [0.24;1.82] | 0.420 |
| **Which substance do you perceive yourself as being most dependent on?** |  |  |
| Tobacco | 1 |  |
| Cannabis | 2.04 [1.03;4.05] | 0.042 |
| Both equally | 1.25 [0.58;2.71] | 0.570 |
| I am not dependent on either substance | 2.74 [1.19;6.32] | 0.018 |
| **Not always mixing cannabis with tobacco when smoked** | 0.77 [0.33;1.80] | 0.551 |
