# Supplementary material for: Simultaneous versus Sequential Cessation of Tobacco and Cannabis: Preferences of Young Adults Who Co‐Use in France
Source: Drug Alcohol Rev. 2026 Aug 9;45(6):e70221. doi: 10.1111/dar.70221 (PMC13453397; doi:10.1111/dar.70221)
Supplement: Supplementary file 8 [file DAR-45-0-s008.docx]

**Supplementary Table 2. Factors associated with preferring simultaneous cessation (alternative logistic regression model, n=357)**

|  | **aOR [95% CI]** | **p-value** |
| --- | --- | --- |
| **Gender** |  |  |
| Men | 1 |  |
| Women | 0.79 [0.48;1.31] | 0.361 |
| Other / Do not want to answer | 0.42 [0.09;1.91] | 0.259 |
| **Age,** in years | 1.01 [0.94;1.09] | 0.684 |
| **Dependence**^1^ |  |  |
| None | 1 |  |
| Tobacco dependence | 0.24 [0.06;0.95] | 0.043 |
| Cannabis dependence | 0.38 [0.19;0.77] | 0.007 |
| Tobacco and cannabis dependence | 0.46 [0.21;1.00] | 0.051 |
| **According to you, based on your current patterns of use, which substance do you think poses the greatest risks to your health (physical or mental)?** |  |  |
| Tobacco | 1 |  |
| Cannabis | 1.28 [0.66;2.47] | 0.470 |
| Both equally | 2.43 [1.33;4.44] | 0.004 |
| I do not know | 0.70 [0.27;1.80] | 0.458 |
| **Which substance do you perceive yourself as being most dependent on?** |  |  |
| Tobacco | 1 |  |
| Cannabis | 1.74 [0.94;3.21] | 0.079 |
| Both equally | 1.71 [0.56;2.47] | 0.678 |
| I am not dependent on either substance | 2.41 [1.07;5.43] | 0.034 |
| **Not always mixing cannabis with tobacco when smoked** | 0.64 [0.29;1.43] | 0.279 |

aOR, adjusted odds ratio; CI, confidence interval; DND, daily or near-daily

^1^ Tobacco dependence is defined as a Fagerström Test for Nicotine Dependence score ≥ 4 (43), and cannabis dependence is defined as a CUDIT-Short Form score ≥ 2 (44)
