# Supplementary material for: Simultaneous versus Sequential Cessation of Tobacco and Cannabis: Preferences of Young Adults Who Co‐Use in France
Source: Drug Alcohol Rev. 2026 Aug 9;45(6):e70221. doi: 10.1111/dar.70221 (PMC13453397; doi:10.1111/dar.70221)
Supplement: Supplementary file 9 [file DAR-45-0-s009.docx]

**Supplementary Table 4. Factors associated with preference for the type of dual cessation (multinomial regression, sensitivity analysis, n=316)**

|  | **Tobacco first vs. simultaneous** |  | **Cannabis first vs. simultaneous** |  | **Cannabis first vs. tobacco first** |  |
| --- | --- | --- | --- | --- | --- | --- |
|  | **aRRR** | **p-value** | **aRRR** | **p-value** | **aRRR** | **p-value** |
| **Gender** |  |  |  |  |  |  |
| Men (ref.) | 1 |  | 1 |  | 1 |  |
| Women | 1.27 [0.68;2.35] | 0.453 | 2.00 [1.05;3.81] | 0.036 | 1.58 [0.87;2.87] | 0.136 |
| Other / Do not want to answer | 6.74 [0.76;59.48] | 0.086 | 3.11 [0.30;32.59] | 0.343 | 0.46 [0.11;1.95] | 0.293 |
| **Age**, in years | 0.96 [0.88;1.05] | 0.381 | 0.98 [0.89;1.08] | 0.707 | 1.02 [0.93;1.12] | 0.638 |
| **Frequency of use** |  |  |  |  |  |  |
| Non-daily tobacco and non-DND cannabis use (ref.) | 1 |  | 1 |  | 1 |  |
| Daily tobacco use, non-DND cannabis use | 0.76 [0.29;2.01] | 0.578 | 1.38 [0.48;4.02] | 0.552 | 1.82 [0.63;5.24] | 0.266 |
| DND cannabis use, non-daily tobacco use | 2.53 [0.89;7.16] | 0.081 | 1.81 [0.49;6.64] | 0.370 | 0.72 [0.21;2.39] | 0.589 |
| Daily tobacco and DND cannabis use | 1.9 [0.83;4.32] | 0.127 | 3.41 [1.32;8.85] | 0.012 | 1.80 [0.73;4.46] | 0.205 |
| **According to you, based on your current patterns of use, which substance do you think poses the greatest risks to your health (physical or mental)?** |  |  |  |  |  |  |
| Tobacco (ref.) | 1 |  | 1 |  | 1 |  |
| Cannabis | 0.71 [0.30;1.67] | 0.428 | 1.70 [0.68;4.25] | 0.253 | 2.41 [1.05;5.57] | 0.039 |
| Both equally | 0.25 [0.12;0.53] | <0.001 | 0.68 [0.32;1.43] | 0.305 | 2.71 [1.24;5.89] | 0.012 |
| I do not know | 1.51 [0.51;4.51] | 0.456 | 1.59 [0.48;5.29] | 0.448 | 1.05 [0.39;2.82] | 0.920 |
| **Which substance do you perceive yourself as being most dependent on?** |  |  |  |  |  |  |
| Tobacco (ref.) | 1 |  | 1 |  | 1 |  |
| Cannabis | 0.69 [0.32;1.49] | 0.345 | 0.32 [0.14;0.74] | 0.008 | 0.46 [0.21;1.01] | 0.052 |
| Both equally | 0.83 [0.33;2.07] | 0.684 | 0.75 [0.30;1.88] | 0.546 | 0.91 [0.40;2.09] | 0.828 |
| I am not dependent on either substance | 0.51 [0.20;1.30] | 0.159 | 0.20 [0.06;0.72] | 0.014 | 0.40 [0.11;1.42] | 0.158 |
| **Not always mixing cannabis with tobacco when smoked** | 1.39 [0.58;3.36] | 0.461 | 1.11 [0.40;3.04] | 0.842 | 0.80 [0.33;1.90] | 0.607 |

aRRR, adjusted relative risk ratio; DND, daily or near-daily

In this analysis, participants receiving care for tobacco or cannabis use (n=41) were excluded.
